# Supplementary material for: Monilinia fructigena Suppressing and Plant Growth Promoting Endophytic Pseudomonas spp. Bacteria Isolated from Plum
Source: Microorganisms. 2022 Dec 3;10(12):2402. doi: 10.3390/microorganisms10122402 (PMC9781308; doi:10.3390/microorganisms10122402)
Supplement: Supplementary file 1 [file microorganisms-10-02402-s001.zip › microorganisms-2044639-supplementary.pdf]

Supplementary Materials

Monilinia fructigena Suppressing and Plant Growth Promoting Endophytic *Pseudomonas* spp. Bacteria Isolated from Plum

Augustina Kolytaitė [1], Dorotėja Vaitiekūnaitė [2], Raminta Antanynienė [1], Danas Baniulis [1,*] and Birutė Frercks [1]

[1] Institute of Horticulture, Lithuanian Research Centre for Agriculture and Forestry, Kaunas Str. 30, Babtai 54333, Kaunas reg., Lithuania; [2] Institute of Forestry, Lithuanian Research Centre for Agriculture and Forestry, Liepu Str. 1, Girionys 53101, Kaunas reg., Lithuania

* Correspondence: danas.baniulis@lammc.lt

Table S1. Physiological test results of biofilm formation, catalase and oxidase production, oxygen requirement and antibiotic susceptibility against ampicillin (AM), cefotaxime (CTX), chloramphenicol (C), kanamycin (K), streptomycin (STP), ticarcillin (TIC).

Morphotype	Sub-morphotypes	Isolate	Antibiotic susceptibility test						Biofilm formation	Catalase test	Oxidase test	Thioglycolate test
			AM-10	CTX-30	C-30	K-30	STP-10	TIC-75				
A	A1	*Pseudomonas graminis*, SENP33	R*	I	I	S	S	R	weak	+	-	Obligate aerobe
		Pseudomonas graminis, SENP33v2	R	I	I	S	S	R	weak	+	-	Obligate aerobe
	A2	*Pseudomonas graminis*, SENP55	R	I	S	S	S	R	moderate	+	-	Obligate aerobe
B	B	*Pseudomonas congelans*, SENP53	I	I	S	S	S	S	weak	+	-	Obligate aerobe
C	C	*Pseudomonas graminis*, SENP58v2	R	S	S	S	S	R	weak	+	-	Obligate aerobe
D	D1	*Pseudomonas amygdali*, SENP41	R	I	S	S	S	S	weak	+	-	Obligate aerobe
	D2	*Agrobacterium fabrum*, SENP78	R	I	I	I	R	I	weak	+	+	Obligate aerobe
		Agrobacterium fabrum, SENP81	R	I	I	I	R	I	weak	+	+	Obligate aerobe

* R – resistant, I – intermediate, S – sensitive.
